# Guanylate-binding protein 2 regulates Drp1-mediated mitochondrial fission to suppress breast cancer cell invasion

**DOI:** 10.1038/cddis.2017.559

**Published:** 2017-10-26

**Authors:** Juan Zhang, Yu Zhang, Wenshuang Wu, Fang Wang, Xinyu Liu, Guanghou Shui, Chunlai Nie

**Affiliations:** 1 Center for Core Facility and Advanced Technologies, Institute of Genetics and Developmental Biology, Chinese Academy of Sciences, Beijing 100101, China; 2 Department of Oncology, Guizhou People’s Hospital, Guizhou 550002, China; 3 State Key Laboratory of Biotherapy and Cancer Center, West China Hospital, Sichuan University and Collaborative Innovation Center for Biotherapy, Chengdu 610041, China; 4 State Key Laboratory of Molecular Developmental Biology, Institute of Genetics and Developmental Biology, Chinese Academy of Sciences, Beijing 100101, China

## Abstract

Guanylate-binding protein 2 (GBP2) is a member of the large GTPase superfamily that is strongly induced by interferon-*γ* (IFN-*γ*). Although the biochemical characteristics of GBP2 have been reported in detail, its biological function has not been thoroughly elucidated to date. To the best of our knowledge, this study presents the first demonstration that GBP2 inhibits mitochondrial fission and cell metastasis in breast cancer cells both *in vitro* and *in vivo*. Our previous work demonstrated that dynamin-related protein 1 (Drp1)-dependent mitochondrial fission has a key role in breast cancer cell invasion. In this study, we demonstrate that GBP2 binds directly to Drp1. Elimination of Drp1 by shRNA or Mdivi-1 (a Drp1-specific inhibitor) suppressed GBP2’s regulatory function. Furthermore, GBP2 blocks Drp1 translocation from the cytosol to mitochondria, thereby attenuating Drp1-dependent mitochondrial fission and breast cancer cell invasion. In summary, our data provide new insights into the function and molecular mechanisms underlying GBP2’s regulation of breast cancer cell invasion.

Guanylate-binding proteins (GBPs) were originally identified as proteins induced by IFN-*γ* in human fibroblasts.^1^ These proteins belong to the superfamily of large GTPases related to dynamin and have similar structural and biochemical properties. Among the seven members of the human GBP family,^2, 3^ GBP1 and GBP2 are mostly induced in cells or tissues upon exposure to IFN-*γ*.^4^ GBP1 can mediate the inhibitory effects of inflammatory cytokines on proliferation, migration and invasion of endothelial cells and epithelial tumor cells.^4, 5, 6, 7, 8^ In addition, GBP1 expression in colorectal carcinoma have been associated with reduced tumor aggressiveness and improved prognosis.^5, 9^ Similarly, GBP2 is postulated as a possible control factor in tumor cell proliferation and spreading.^10, 11^ Increased GBP2 expression is also associated with a better prognosis in breast cancer and may have a role in T-cell defense against breast cancer.^12^ Interestingly, GBP2 expression in NIH 3T3 fibroblasts inhibited Rac activation and matrix metalloproteinase-9 expression,^13^ suggesting a possible role for GBP2 in regulating cancer metastasis. However, the targets and molecular mechanisms of GBP2’s regulation of cancer metastasis remain largely unknown.

Mitochondria exist as dynamic networks maintained by two opposing processes: fission and fusion,^14^ primarily regulated by dynamin-related protein 1 (Drp1) and mitofusins (Mfns),^14, 15^ respectively. Mitochondrial fission or fusion dysfunction deregulates key cellular processes, potentially contributing to tumorigenesis.^16^ We previously reported that Drp1-dependent mitochondrial fission is critical for breast cancer cell invasion.^17^ As breast cancer metastasis is a highly complex process regulated by many factors, we reason that there may be additional factors participating in mitochondrial dynamics that regulate breast cancer metastasis. In the present study, we found that GBP2 interacts with Drp1 and blocks translocation of Drp1 to mitochondria, thereby attenuating Drp1-dependent mitochondrial fission and invasion of breast cancer cells. Thus GBP2 may represent a new therapeutic target to suppress breast cancer metastasis through attenuation of Drp1-dependent mitochondrial fission.

## Results

### GBP2 expression inhibits invasion and mitochondrial elongation in breast cancer cells

**Figure 1:**
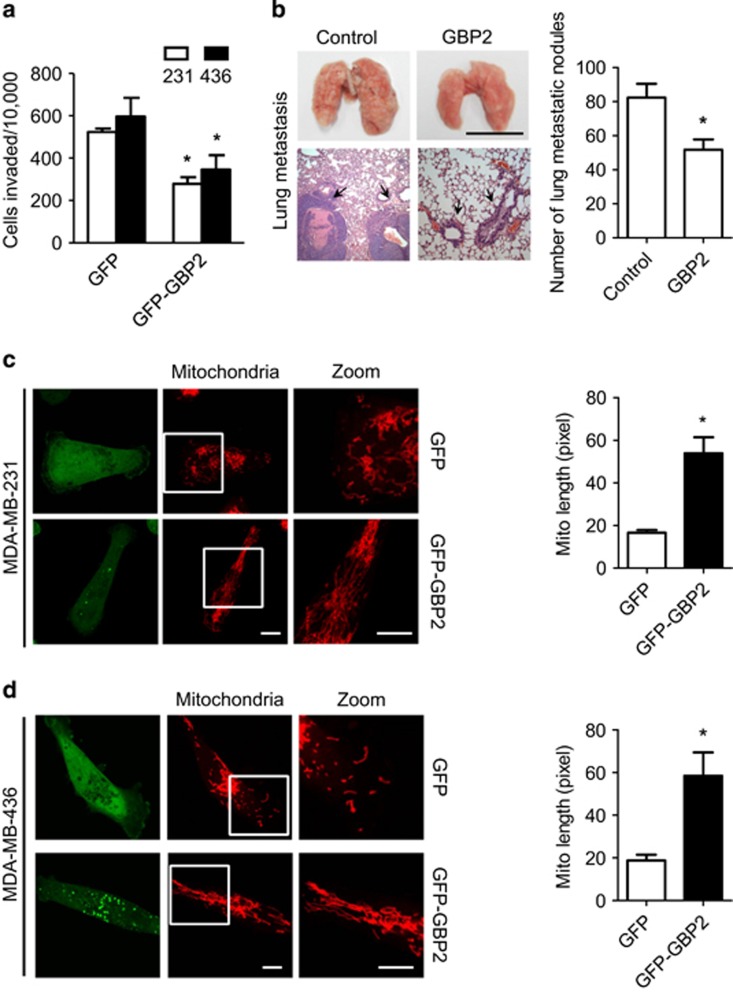
Expression of GBP2 inhibits invasion and induces mitochondrial elongation of breast cancer cells. (**a**) Overexpression of GFP-tagged GBP2 inhibited invasion of breast cancer MDA-MB-231 and MDA-MB-436 cells. Data shown are mean±S.E.M. (*n*=4), *P<0.05. (**b**) Upregulation of GBP2 in MDA-MB-231 cells decreases the metastasis ability in mice. Left panel: Upper is the representative imaging of lung tissues from mouse model of metastasis. Bars, 1 cm; Below is the representative hematoxylin and eosin–stained lung sections exhibiting metastasis in representative mice, magnification of the histopathological sections, × 50. Right panel: Nodules rich in densely packed cells, as indicated by the black arrows, were quantified as tumor nodules. Values represent the mean±S.E.M. (n=8-11), *P<0.05. The representative images of (**c**) MDA-MB-231 cells and (**d**) MDA-MB-436 cells expressing GFP or GFP-GBP2 (green). Mitochondria were visualized with MitoTracker Red. Right panel is the quantification of mitochondrial lengths in MDA-MB-231 and MDA-MB-436 cells. Scale bar, 10 *μ*m. Data shown are mean±S.E.M. *n*=29–42 randomly selected cells, *P<0.05

To investigate whether GBP2 modulates cancer metastasis, GFP-tagged GBP2 or GFP vector were transfected into the indicated metastatic breast cancer cells (Supplementary Figure 1a). Transwell assays^18^ demonstrated that cells expressing GFP-tagged GBP2 exhibited a decrease in invasive abilities compared with their control cells expressing GFP alone (Figure 1a). Moreover, animal experiments revealed that most control mice with mammary tumors developed massive lung metastases, but GBP2-expressing mice had significantly fewer lung metastases (Figure 1b). Interestingly, GBP2 expression did not increase apoptosis or cell death (Supplementary Figures 1b and c). The expression of GBP1 had little effect on cell invasion under the same conditions (data not shown).

Given that breast cancer cell metastasis can be regulated by mitochondrial dynamics, we tested whether GBP2 could alter mitochondrial networks. As shown in Figures 1c and d (left panels), GFP was distributed throughout the cytoplasm, whereas tagged GBP2 was distributed in specific subcellular regions, as described before.^19^ Expression of GBP2 led mitochondria to be more filamentous and increased mitochondrial length in the indicated cells (Figures 1c and d, right panel). We also transfected GFP or GFP-tagged GBP1 vector into the indicated cells to see whether GBP1 affects mitochondrial dynamics. We found that GBP1 expression had little effect on mitochondrial elongation (Supplementary Figure 2a).

**Figure 2:**
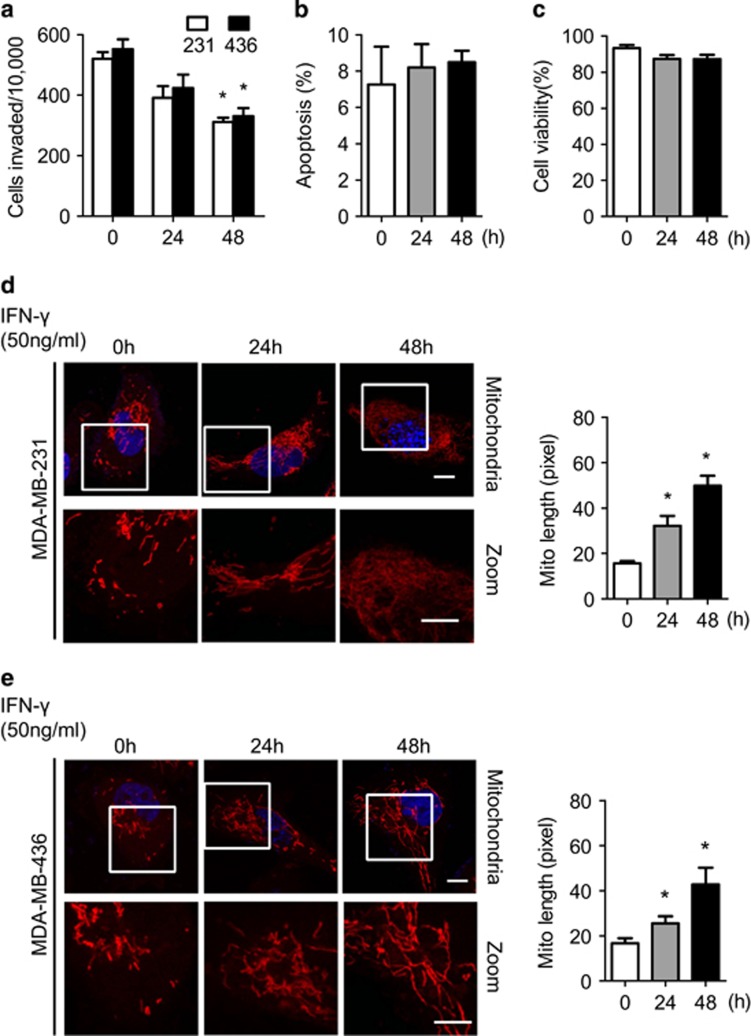
IFN-*γ* treatment led to inhibition invasion and mitochondrial elongation of breast cancer cell. (**a**) IFN-*γ* (50 ng/ml) treatment for 24 and 48 h inhibits invasive abilities of breast cancer MDA-MB-231 and MDA-MB-436 cells. Data shown are mean±S.E.M. (*n*=4), *P<0.05. (**b**) IFN-*γ* treatment for 24 and 48 h could not induce cell apoptosis and (**c**) initiates cell viability in MDA-MB-231 cells. (**d**) MDA-MB-231 and (**e**) MDA-MB-436 cells were treated with 50 ng/ml IFN-*γ* for 24 and 48 h. Left panel, Cells were stained with Mitotracker Red and visualized under confocal microscope. Scale bar, 10 *μ*m. Right panels, Quantification of mitochondrial lengths. Data shown are mean±S.E.M., *P<0.05. *n*=19–29 randomly selected cells

We then used IFN-*γ* to induce endogenous GBP2 expression in cells.^2^ As shown in Supplementary Figure 2b, IFN-*γ* efficiently induced GBP2 expression in cells. Furthermore, Figure 2a shows that treatment with IFN-*γ* reduced invasion of the indicated cells. It should be noted that IFN-*γ* treatment at a certain concentration (50 ng/ml) did not result in the change of cell apoptosis (Figure 2b) or cell viability (Figure 2c) in cells.

Confocal images show that treatment with recombinant IFN-*γ* resulted in time-dependent mitochondrial elongation in the indicated cells (Figures 2d and e, left panels). The average length of mitochondria was increased after IFN-*γ* treatment (Figures 2d and e, right panels).

**Figure 3:**
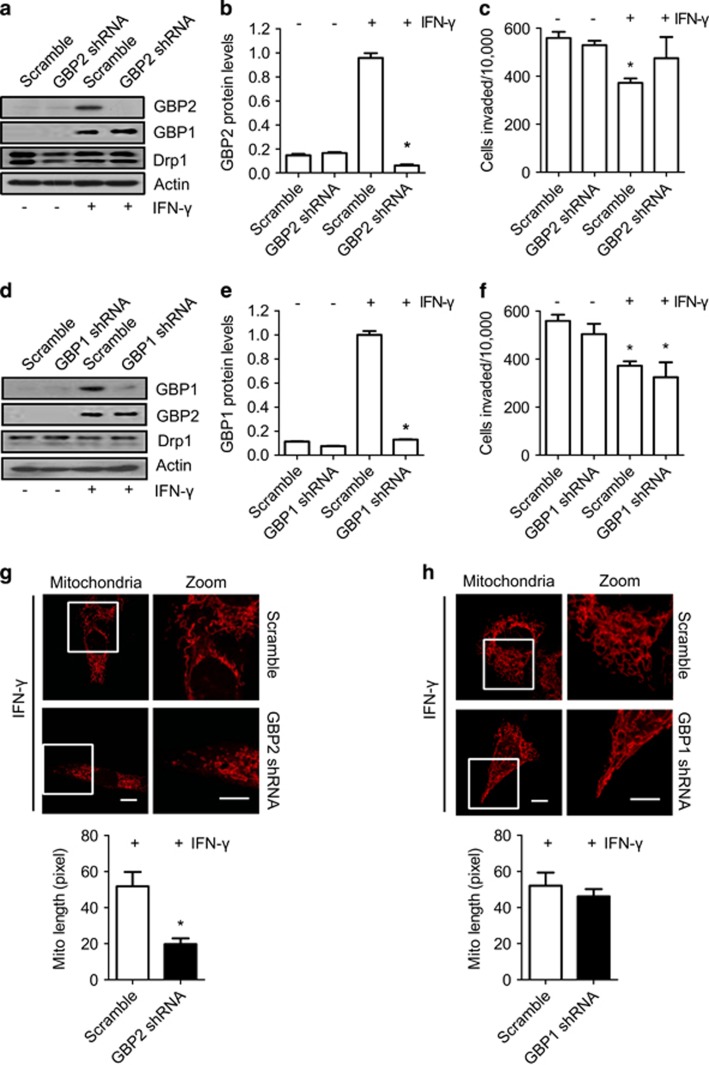
GBP2 is essential for IFN-*γ*-induced mitochondrial elongation and inhibition of breast cancer cell invasion. (**a**) IFN-*γ*-induced GBP2 in MDA-MB-231 cells was depleted by GBP2 shRNA but not scramble shRNA. A blot representative of three experiments is shown. (**b**) The histogram shows the mean GBP2 band density, which was measured and normalized to *β*-actin±S.E.M., *n*=3, *P<0.05. (**c**) GBP2 depletion abolished IFN-*γ*-induced inhibition of MDA-MB-231 cell invasion. Data shown are mean±S.E.M. (*n*=4), *P<0.05. (**d**) GBP1 in MDA-MB-231 was depleted by GBP1 shRNA but not scramble shRNA. A blot representative of three experiments is shown. (**e**) The histogram shows the mean GBP1 band density, which was measured and normalized to *β*-actin±S.E.M., *n*=3, *P<0.05. (**f**) GBP1 depletion had little effect on IFN-*γ*-induced inhibition of MDA-MB-231 cell invasion. Data shown are mean±S.E.M. (*n*=4). (**g**) Upper panel, the representative images of MDA-MB-231 cells transfected with the pSliencer-2.1 null vector or vector expressing GBP2 shRNA. Mitochondria were visualized with MitoTracker Red. Scale bar, 10 *μ*m. Lower panel, data shown in the upper panel are the average mitochondrial lengths in 19–22 randomly selected MDA-MB-231 cells. Error bars, S.E.M., *P<0.05. (**h**) Upper panel, the representative images of MDA-MB-231 cells transfected with the pSliencer-2.1 null vector or vector expressing GBP1 shRNA. Scale bar, 10 *μ*m. Lower panel, data shown in the upper panel are the average mitochondrial lengths in 19–22 randomly selected MDA-MB-231 cells. Error bars, S.E.M., *P<0.05

As many proteins respond to IFN-*γ* stimulation, we needed to determine whether the effects of IFN-*γ* on invasion and mitochondrial dynamics in breast cancer cells were dependent on induction of GBP2, rather than other inducible proteins. We next transfected the indicated cells with GBP2 shRNA to deplete IFN-*γ*-induced GBP2 (Figures 3a and b). Inhibition of GBP2 expression restrained the effect of IFN-*γ* on the invasive abilities of cells (Figure 3c). GBP1 protein was also expressed in the indicated cells with IFN-*γ* treatment (Figures 3d and e).^1^ GBP1 shRNA in the indicated cells efficiently reduced GBP1 expression in response to IFN-*γ* treatment. However, GBP1 depletion had little effect on the invasive abilities of the treated cells (Figure 3f). Moreover, GBP2 depletion abolished IFN-*γ*-induced mitochondrial elongation (Figure 3g), while GBP1 depletion failed to change elongated mitochondria induced by IFN-*γ* (Figure 3h). Taken together, our data suggest that GBP2 specifically reduces invasion and is involved in regulating mitochondrial dynamics in metastatic breast cancer cells.

### Drp1 is a cellular binding factor of GBP2

**Figure 4:**
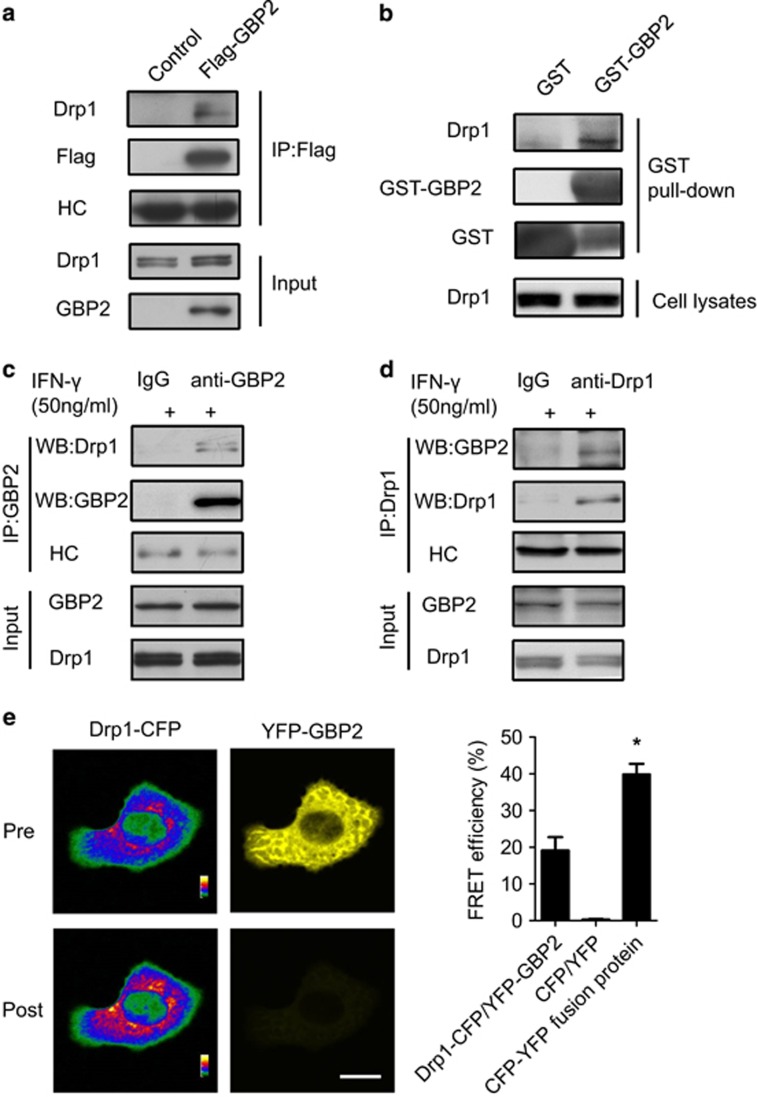
Identification of Drp1 as a GBP2 interaction protein in breast cancer cells. (**a**) Immunoprecipitation of the extracts from control MDA-MB-231 cells or cells expressing Flag-tagged GBP2 using anti-Flag antibody, followed by western blotting analysis with antibodies against the Flag or Drp1. Heavy chain (HC) of anti-Flag antibody was used as a loading control. Rabbit IgG was used as a control. The presence of GBP2 and Drp1 was detected by western blotting. (**b**) Western blotting analysis of GST and GST-GBP2 precipitates of MDA-MB-231 cell lysates using anti-Drp1 or anti-GST antibody. (**c** and **d**) IFN-*γ*-induced GBP2 interacts with Drp1 in MDA-MB-231 cells. Co-immunoprecipitation assays of IFN-*γ*-treated MDA-MB-231 cell lysates using anti-GBP2 (**c**) or anti-Drp1 antibody (**d**). (**e**) Left panel, fluorescent images of MDA-MB-231 cells co-transfected with Drp1-CFP and YFP-GBP2 in CFP and YFP channels prephotobleaching and postphotobleaching of YFP fluorescence. Scale bar, 10 *μ*m. Right panel, The histogram shows the FRET efficiency from CFP to YFP in the cells transfected with Drp1-CFP and YFP-GBP2, CFP and YFP or CFP–YFP fusion protein. Data shown are mean±S.E.M. (*n*=15), *P<0.05

Next, we characterized the molecular mechanism of GBP2’s participation in cell invasion and mitochondrial dynamics. Previous work demonstrated that Drp1-dependent mitochondrial fission regulates metastasis of breast cancer cells.^17^ We found that IFN-*γ* treatment resulted in mitochondrial elongation and induction of GBP2 expression, with little change in Drp1 expression or Mfn1 and Mfn2 in the indicated cells (Supplementary Figure 2b). It is possible that GBP2 interacts with Drp1. To test this hypothesis, we first performed co-immunoprecipitation assays to identify whether GBP2 can bind to Drp1 in whole-cell extracts of cells. As low expression levels of endogenous GBP2 in cells (Supplementary Figure 2b) would make it difficult to detect an interaction between GBP2 and Drp1, we employed exogenous expression of GBP2 as well as IFN-*γ* treatment to induce endogenous GBP2. Indicated cells were transfected with Flag-GBP2 constructs. Co-immunoprecipitation revealed the presence of Drp1 in the Flag-GBP2 immunoprecipitate (Figure 4a). Meanwhile, Drp1 failed to co-precipitate with Flag-GBP1 (Supplementary Figure 2c). We also performed GST-GBP2 pull-down assays in the indicated cells. GST-GBP2 pull-down assays combined with western blotting analysis showed the presence of Drp1 in the pull-down fraction of GST-GBP2 but not in the GST control (Figure 4b). We then performed GST-GBP2 pull-down assays using the indicated cell lysates combined with mass spectrometric analysis. Drp1 was indeed identified in GST-GBP2 precipitate but not in control samples in two independent mass spectrometric experiments (Supplementary Figures 3a and b). Co-immunoprecipitation assays with IFN-*γ*-treated cell lysates showed the presence of Drp1 in the GBP2 immunoprecipitate (Figure 4c) and the presence of GBP2 in the Drp1 immunoprecipitate (Figure 4d). In contrast, no GBP2 or Drp1 was precipitated when rabbit IgG was used. Together, these results suggest that GBP2 interacts with endogenous Drp1 in breast cancer cells.

To validate our findings that GBP2 interacts physically with Drp1, we performed fluorescence resonance energy transfer (FRET) analysis as described before^20^ to validate whether GBP2 interacts directly with Drp1. Indicated cells were co-transfected with Drp1-CFP and YFP-GBP2, and the fluorescent intensity of the donor Drp1-CFP or the acceptor YFP-GBP2 was measured before and after photobleaching by a laser beam. The fluorescent intensity of Drp1-CFP was remarkably increased after the fluorescence of YFP-GBP was completely quenched (Figure 4e, left panel). The energy transfer efficiency from Drp1-CFP to YFP-GBP2 was 19.1±3.7% (Figure 4e, right panel), suggesting a direct interaction between Drp1-CFP and YFP-GBP2. In contrast, little energy transfer was detected in cells co-transfected with CFP and YFP proteins. As a positive control, the energy transfer efficiency was 40.3±2.8% within a CFP-YFP fusion protein. Collectively, our data demonstrate that Drp1 and GBP2 have a physical interaction in breast cancer cells.

Previous reports have shown that phosphorylation of Ser 637 in Drp1 promotes mitochondrial elongation.^21, 22^ However, the phosphorylation of Ser 616 promotes mitochondrial fragmentation.^23, 24^ To identify whether GBP2 has a role in regulating phosphorylation of Drp1, exogenous GBP2 was expressed in the indicated cells, and the phosphorylation of Drp1 was determined by western blotting. As shown in Supplementary Figures 3c and d, GBP2 had little effect on either Ser 637 or Ser 616 phosphorylation status of Drp1, suggesting that GBP2 does not regulate the function of Drp1 by modulating its phosphorylation.

### GBP2 regulation of cell invasion and mitochondrial fission depends on Drp1

**Figure 5:**
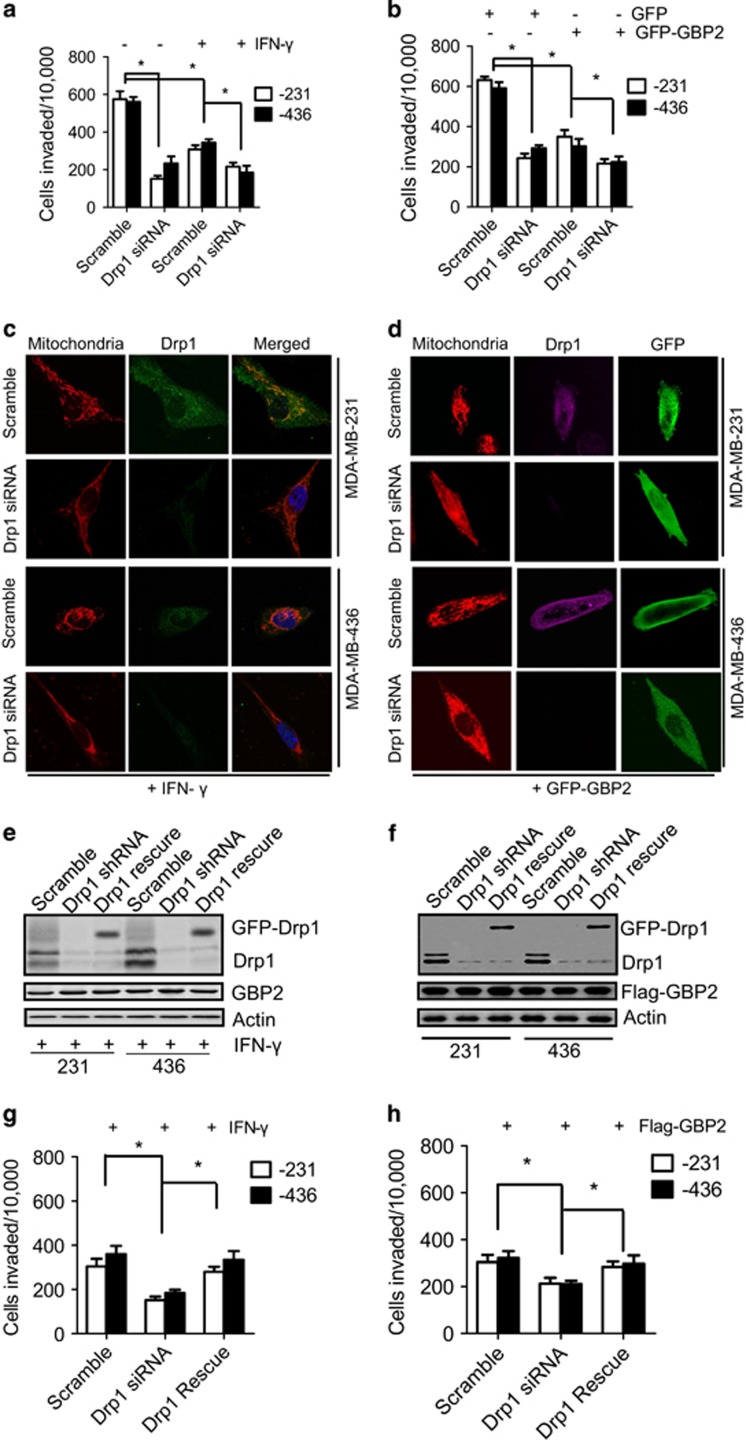
Drp1 depletion inhibits GBP2 mediated cell invasion and mitochondrial fission. Knockdown of endogenous Drp1 inhibits invasion abilities of breast cancer MDA-MB-231 and MDA-MB-436 cells with (**a**) IFN-*γ* treatment or (**b**) transfected with GFP-GBP2 or GFP as control. The histogram shows cell invasion. Data shown are mean±S.E.M. (*n*=4), *P<0.05. (**c** and **d**) Representative confocal images of MDA-MB-231 cells (upper) and MDA-MB-436 cells (lower) with IFN-*γ* treatment or GFP-GBP2 expression, transfected with scramble or Drp1 shRNA and stained with MitoTracker Red, show endogenous expression of Drp1 and mitochondrial morphology; green shows exogenous GBP2 expression. Scale bar, 10 mm. (**e** and **f**) A GFP-tagged Drp1 mutant, insensitive to Drp1 shRNA, was expressed in Drp1-silenced breast cancer cells with IFN-*γ* treatment or Flag-GBP2 expression for 48 h, and cells were then collected for western blotting analysis of Drp1 expression. (**g** and **h**) As described in panels (**e** and **f**), cells were collected for transwell invasion assays. *n*=4, mean±S.E.M. *P<0.05

Next, we addressed the biological significance of the interaction between GBP2 and Drp1. Depletion of Drp1 with shRNA was utilized to see whether it blocks cell invasion regulated by GBP2. We found that Drp1 shRNA had little effect on GBP2 expression in the indicated cells treated with IFN-*γ* or overexpression of GBP2 (Supplementary Figures 4a–d). However, it was noteworthy that Drp1 depletion reduced invasion in cells treated with IFN-*γ* or overexpression of GBP2 (Figures 5a and b). Meanwhile, Drp1 depletion decreased mitochondrial fission and promoted elongation of cells regardless of IFN-*γ*-induced GBP2 expression or overexpression of GBP2 (Figures 5c and d). To rule out ‘off-target’ effects of shRNA, we next carried out rescue experiments by re-expressing GFP-tagged Drp1 with a mutation that is insensitive to Drp1 shRNAs in Drp1-silenced cells (Figures 5e and f). GFP-Drp1 efficiently restored invasion of the Drp1-silenced cells with GBP2 expression induced by IFN-*γ* (Figure 5g) or transfected with Flag-GBP2 (Figure 5h). These results suggest that GBP2 is an upstream regulator of Drp1-dependent cell invasion and regulates cell invasion and mitochondrial fission through Drp1.

To determine GBP2 dependence on Drp1 to regulate cell invasion of breast cancer cells, we treated cells with Mdivi-1, a Drp1-specific inhibitor that allows for unopposed fusion.^25^ As shown in Supplementary Figure 4e, Mdivi-1 treatment destroyed GBP2-Drp1 binding and inhibited invasion of cells with or without IFN-*γ*-induced GBP2 expression (Supplementary Figure 4f). Moreover, GBP2 expression was synergistic with Mdivi-1 in further reducing cell invasion, which suggests that both GBP2 and Mdivi-1 regulate cell invasion through targeting Drp1. These results further indicate that GBP2-regulated cell invasion and mitochondrial fission is dependent on Drp1.

### GBP2 K51A mutation blocks interaction with Drp1 and inhibition of mitochondria fission

**Figure 6:**
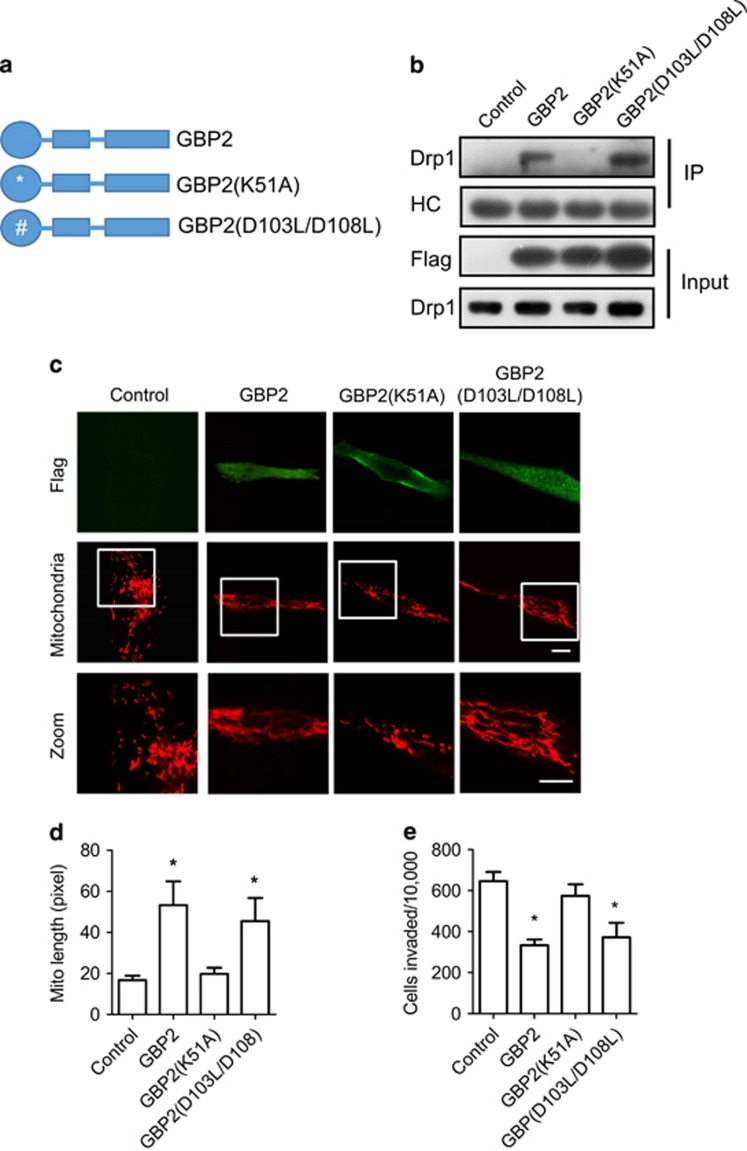
Domains of GBP2 are required for its interaction with Drp1 and inhibition of mitochondria fission. (**a**) Panel of GBP2 and its mutants. GBP2^D103L/D108L^, defective in hydrolysis from GTP to GMP; GBP2^K51A^, GTPase-defective mutant. (**b**) Cell lysates of MDA-MB-231 cells expressing Flag-GBP2, GBP2 mutants or empty vector as indicated were subjected to immunoprecipitation with anti-Flag antibody, followed by western blotting with Drp1 antibody. The heavy chain of immunoglobulins was used as loading control. (**c**) The confocal images of MDA-MB-231 cells expressing Flag-tagged GBP2, GBP2 mutants or empty vector. Mitochondria were visualized with Mitotracker red. Scale bar, 10 *μ*m. (**d**) The length of mitochondria was quantified using Image-Pro Plus software. Data shown are mean±S.E.M. of 25–30 randomly selected cells, *P<0.05. (**e**) The histogram shows cell invasion of MDA-MB-231 cells expressing Flag-tagged GBP2, GBP2 mutants or empty vector. Data shown are mean±S.E.M. (*n*=4), *P<0.05

To determine which structural domain of GBP2 is responsible for the interaction of GBP2 with Drp1, several truncated or mutated GBP constructs with a Flag tag (Figure 6a,Supplementary Figure 5a) were generated and expressed in the indicated cells. The interaction of these proteins with endogenous Drp1 was examined by Co-IP experiments using anti-Flag antibody. As shown in Supplementary Figure 5b, deletion of N-terminal GTPase globular domain (GBP2^276–591^), C-terminal helical domain (GBP2^1–308^) or N-terminal GTPase globular and middle connecting domains (GBP2^308–591^)^26^ diminished GBP2 interaction with Drp1, indicating that the integrated structure of GBP2 is requisite for its interaction with Drp1. However, it is interesting that GBP^K51A^, a GTPase-defective mutant,^27^ could inhibit interaction with Drp1, while GBP2^D103L/D108L^, another GTPase-defective mutant,^26, 28^ could still effectively interact with Drp1 (Figure 6b).

Further experiments revealed that overexpression of full-length GBP2 or the GBP2^D103L/D108L^ mutant led to elongation of mitochondria in cells (Figures 6c and d). In contrast, GBP2^K51A^ expression had little effect on the length of mitochondria in cells, as well as the expression of GBP2^276–591^, GBP2^1–308^ or GBP2^308–591^ mutants (Supplementary Figure 5c). Moreover, the expression of GBP2^K51A^ mutant significantly rescued cell invasion, compared with the expression of GBP2 or the GBP2^D103L/D108L^ mutant (Figure 6e). GBP2^276–591^, GBP2^1–308^ or GBP2^308–591^ mutant expression failed to block cell invasion (Supplementary Figure 5d).

These data reveal that Lysine 51 of GBP2 is critical for the interaction between GBP2 and Drp1, and every domain of GBP2 participates in binding with Drp1.

### GBP2 blocks Drp1 translocation to the mitochondria and inhibits mitochondrial fission and cell invasion

**Figure 7:**
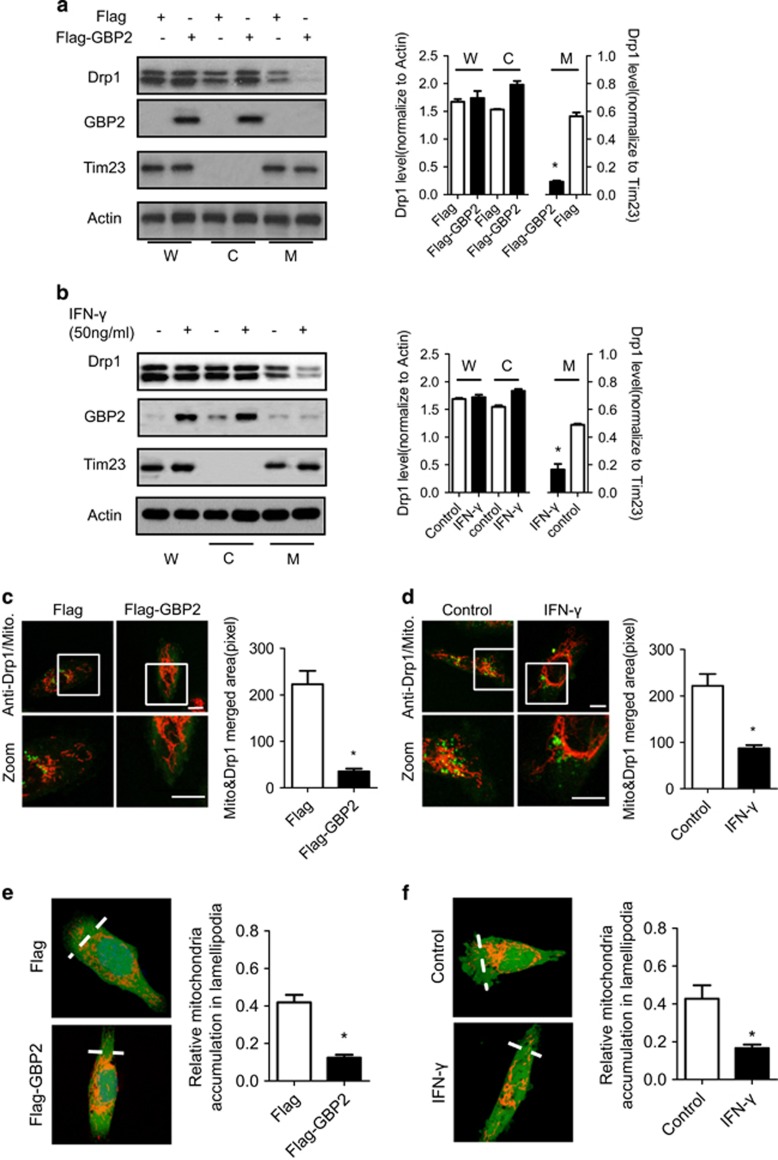
GBP2 blocks Drp1 translocation from cytosol to mitochondria and the distribution to lamellipodia. Western blotting analysis of distribution of Drp1 in the mitochondrial fraction in (**a**) MDA-MB-231 cells expressing Flag or Flag-tagged GBP2 or (**b**) cells pretreated without or with IFN-*γ* (50 ng/ml) for 48 h. Equal amounts of protein of whole-cell lysate (W), cytosol (C) and mitochondrial (M) fractions were loaded on SDS-PAGE and analyzed by western blotting using anti-Drp1 and GBP2 antibodies. Right panel, Drp1 band density was measured and normalized to *β*-actin (W or C) or Tim23 (M), Mean± s.e.m (*n*=3). *P<0.05 between two groups. *β*-Actin was used as a loading control of whole cell lysate and cytosol fraction whereas Tim23 was used as the loading control of mitochondrial fraction. (**c** and **d**) Confocal microscopic images showing the subcellular distribution of endogenous Drp1 in MDA-MB-231 cells transfected with Flag-GBP2 or Flag protein (**c**) and MDA-MB-231 cells treated without or with IFN-*γ* (**d**). Cells were stained with Mitotracker Red and then immuno-labelled with anti-Drp1 antibody (green). Scale bar, 10 *μ*m. The histograms show the statistics of co-localization level of drp1 and mitochondria in panels (**c** and **d**). Data shown are mean±S.E.M., *P<0.05, *n*=18–25 randomly selected cells. (**e** and **f**) Upregulation of GBP2 block mitochondrial distribution to lamellipodia in MDA-MB-231 cells induced by Chemoattractant NIH-3T3 CM. MDA-MB-231 cells stained with MitoTracker Red and CellTracker Green were visualized with a confocal microscope and the integrated fluorescent intensity was analyzed by the Image-Pro Plus software. The relative abundance of mitochondria in the lamellipodia region was calculated as described in Materials and Methods. Columns, means; bar, S.E.M. *P<0.05. *n*=45–52 randomly selected cells

It has been shown that translocation of Drp1 to the mitochondria is critical for its regulation of mitochondrial fission.^29^ It is possible that GBP2 suppresses mitochondrial fission and cell invasion via regulation of Drp1 translocation to mitochondria. To test this hypothesis, we performed subcellular fractionation assays using lysates of the indicated cells transfected with vector encoding Flag-tagged GBP2 or just the Flag tag. Western blotting shows that GBP2 is located predominately in the cytosol. There was no detectable GBP2 in the mitochondrial fraction, as described before.^19^ Drp1 was mainly located in the cytosol and marginally in the mitochondrial fraction (Figure 7a, left panel), as described before.^30^ Compared with control Flag, overexpression of Flag-tagged GBP2 decreased the amount of Drp1 protein in the mitochondrial fraction by approximately 70%, whereas the Drp1 level in the cytosolic fraction was increased (Figure 7a, right panel). Similarly, IFN-*γ* treatment induced GBP2 expression and significantly decreased the amount of Drp1 protein in the mitochondrial fraction by >50% (Figure 7b). In contrast, there was no change in the amount of Mfn1/2 in the mitochondrial fraction (data not shown). Meanwhile, the GBP2 mutant, GBP^K51A^, which fails to bind Drp1, had little effect on Drp1 translocation (Supplementary Figure 6a) or mitochondrial fission and cell invasion (Figures 6d and e). Moreover, confocal microscopy revealed that upregulation of GBP2 by exogenous expression of GBP2 or IFN-*γ* treatment altered subcellular localization of Drp1 (Figures 7c and d, left panel). Immuno-staining analysis shows that, in control cells or cells transfected with Flag protein, Drp1 (green) was distributed as puncta, often localized on mitochondrial tubules (indicated by MitoTracker red) (Figure 7c), and a similar observation was made in IFN-*γ*-treated cells (Figure 7d). This punctate Drp1 staining represents Drp1 protein localized at the mitochondria, which is required for mitochondrial fission. In contrast, the punctate staining of Drp1 on mitochondrial tubules was decreased in GBP2-overexpressed or IFN-*γ*-treated cells by 84% and 60%, respectively (Figures 7c and d, right panel). To determine whether decreased Drp1 at the mitochondria was due to upregulation of GBP2 expression, we transfected Flag-GBP2 (green) into MDA-MB-231 cells and performed immuno-staining to detect the change in Drp1 translocation. As shown in Supplementary Figure 6b, the distribution of endogenous Drp1 (red) changed from partially localized within the mitochondria (white) to clustered and partially co-localized with GBP2 (green) and restricted in the cytoplasm, along with mitochondrial morphology changing from tubular to more filamentous, consistent with our other data.

To ascertain the effect of GBP2 on Drp1 translocation, we co-transfected GBP2 and Drp1 constructs into MCF-7 cells. We used MCF-7 cells because our previous study reported that Drp1 expression levels were low in non-metastatic breast cancer cell lines, such as MCF-7 cells.^17^ Moreover, endogenous GBP2 expression was also low in MCF-7 cells (data not shown). Confocal images show that control cells have filamental mitochondria (Supplementary Figure 6c, left panel). Myc-tagged Drp1 expression (green) showed partial co-localization with mitochondria as puncta and induced mitochondrial fragmentation in cells. Interestingly, co-expression of Flag-tagged GBP2 changes the distribution of myc-tagged Drp1 (green). The majority of Drp1 was found to be co-localized with Flag-GBP2 (purple) near the edge of the cytoplasm with little on mitochondrial tubules (Supplementary Figure 6c, green panel). As expected, co-expression of GBP2 attenuated Drp1-induced fission of mitochondria (Supplementary Figure 6c, red panel).

Previous work revealed that mitochondrial fission directs mitochondrial accumulation to the lamellipodia region at the leading edge of cells. We speculate that the effect of GBP2 on Drp1 translocation also blocks mitochondrial accumulation at the leading edge of cells. As shown in Figures 7e and f, mitochondrial localization changes during GBP2 upregulation were quantified,^17^ and overexpression of GBP2 or IFN-*γ* treatment reduced mitochondrial accumulation in the lamellipodia region.

Collectively, our data indicate that GBP2 attenuates subcellular localization of Drp1 to mitochondria through interaction with Drp1 in the cytosol, suppressing mitochondrial fission and cell invasion.

## Discussion

We previously reported that Drp1-dependent mitochondrial fission regulates breast cancer cell migration and invasion.^17^ In this study, GBP2 was found to bind directly to Drp1 and block Drp1 translocation from the cytoplasm to the mitochondria, which is a potential mechanism for reducing mitochondrial fission. The interaction between GBP2 and Drp1 was systematically and consistently confirmed under various experimental conditions using both ectopically and endogenously expressed proteins. As an IFN-*γ*-inducible protein, GBP2 belongs to the larger dynamin superfamily of GTPases that include, for instance, Dynamin and Mx. GBP2 can oligomerize with itself or with other GBPs.^23^ GBP2 was previously described to bind GBP1 and to polymerize around cytosolic bacteria to allow escape from cytosolic bacteria through autophagy.^31^ However, we demonstrate that Drp1, another kind of dynamin family protein, is a new target for GBP2. Despite limited sequence homology, GBP2 and Drp1 share similar biochemical features,^29^ such as the conserved GTPase domain. Moreover, the crystal structure of GBP^32, 33^ intramolecular interactions was found to be similar to those that have been predicted for dynamins, such as assembled Drp1. It is possible that GBP2 and Drp1 bind to each other to form hetero-oligomers.

To be oligomerized, GBP family proteins first need the N-terminal GTPase domains to self-dimerize, which requires GTPase activity. Enzymatic activity of GBP leads to a structural shift, making previously buried sites on the C-terminal domain available for further interaction.^26, 34^ Thus GTPase activity is necessary for the oligomerization of GBP family proteins, as well as for dynamin superfamily proteins.^27, 26^ In our study, two GTPase-defective mutants, GBP^K51A^ and GBP^D103L/D108L^, have different effect on Drp1 binding. GBP^K51A^ failed to interact with Drp1, while GBP2^D103L/D108L^ could bind Drp1. As previously reported, GBP1 and GBP2 share 75% sequence homology and have the same domains for GTPase and dimerization.^26^ We can learn from GBP1 to study the function of GBP2. GBP2^K51A^ or GBP1^K51A^ mutant was reported to strongly decrease the affinity of all three mant-nucleotides (mant-GTP, mant-GDP and mant-GMP), indicating that the GBP^K51A^ mutants will occur as predominantly nucleotide-free.^35^ Moreover, the GBP2^K51A^ mutant was constitutively monomeric, and the GTPase hydrolytic activity of GBP2^K51A^ mutant was negligible.^35, 36^ GBP1^D103L/D108L^ was a monomer with nucleotides. The double mutant could only hydrolyze GTP to GDP and lacked the second hydrolysis activity, suggesting that the GBP^D103L/D108L^ mutant could bind with GTP and GDP.^26, 28^ Thus the GBP2^K51A^ and GBP2^D103L/D108L^ mutants may have two defects, differences in nucleotide affinity and hydrolytic activity. First, the GBP2^K51A^ mutant is nucleotide-free; GBP2^D103L/D108^ mutants are not and can bind with GTP and GDP. Second, GBP2^K51A^ has little GTP hydrolysis; the double mutants have only the first hydrolytic activity. It is noteworthy that the ^103^DXEKGD^108^ motif, including D103 and D108, is located at the dimeric interface of GBP1, and the D103 and D108 residues have not been reported to interact in dimerization of GBP1.^28, 37^ Furthermore, the ^103^DXEKGD^108^ motif of GBP1 is unique for GBP family proteins and not found in other GTP-binding proteins.^26^ These reports indicated the K51 residue is more important for the dimerization of different groups of dynamin proteins. After all, immunofluorescence assays revealed that the GBP2^K51A^ mutant was distributed evenly in the cytoplasm, which is consistent with a previous report,^27^ whereas GBP2 wild type or GBP2^D103L/D108L^ mutant was distributed with granular accumulation in the cytosol. These results further suggest that K51 and the ^103^DXEKGD^108^ motifs have different roles in GBP2’s interaction with Drp1.

A recent study revealed that high GBP2 is associated with better prognosis in breast cancer, particularly in quickly proliferating tumors.^12^ Further testing predicted that GBP2 correlates with T-cell signature, indicating tumor infiltration with T cells. In our study, we demonstrated that upregulation of GBP2 expression suppresses metastasis in breast cancer cells by inhibiting Drp-1-dependent mitochondrial fission, providing further understanding of the role and mechanism of GBP2 in breast cancer. We used MDA-MB-231, MDA-MB-436 (estrogen receptor (ER) negative, metastatic) and MCF-7 (ER positive, non-metastatic) cells^17, 38^ for our *in vitro* models. Drp1 expression is high in metastatic breast cancer, and clinical findings have demonstrated that ER-negative carcinomas usually proliferate faster^12^ and are more inclined to nodal metastasis than are ER-positive neoplasms.^39^ Thus high Drp1 expression in breast tumors may be promoting high rates of proliferation and metastasis. High GBP2 expression could inhibit Drp1-mediated breast cancer invasion and reduce the risk of tumor recurrence. After all, the risk of recurrence in patients with ER-negative primary cancer is higher than that in ER-positive tumors.^12^ Because there are many subtypes of breast cancer based on ER, HER and PR status, we still need to determine the expression and role of GBP2 and Drp1 in additional cancer specimens to determine whether the role of GBP2 and Drp1 in metastasis and tumorigenesis is conserved in other breast cancer subtypes.

We next tested increased GBP2 levels to determine the function in breast cancer cells with low GBP2 expression and found that exogenous GBP2 transfection led to a greater inhibition of metastasis in cells than did IFN-*γ* treatment (Figure 1a,Figure 2a). This data supports the finding that high GBP2 levels are associated with improved metastasis-free survival in node-negative breast carcinomas.^12^ It should be noted that increasing IFN-*γ* concentration failed to further increase GBP2 expression in cells (Supplementary Figure 7b) but increased expression of other proteins that promote breast cancer metastasis.^40^ This may provide an additional explanation for why IFN-*γ* is not an efficient breast cancer therapy. Thus targeting GBP2, an IFN-*γ* inducible protein, represents a novel approach for suppressing breast cancer invasion.

In conclusion, out data show for the first time that GBP2 directly interacts with Drp1, and we identify GBP2 as an inhibitory factor in breast cancer metastasis. Our data suggest that upregulation of GBP2 expression, a key step to block Drp1-dependent mitochondrial fission, may represent a novel strategy to prevent metastasis in breast cancers.

## Materials and methods

### Cell culture and transfection

All cell lines were from the American Type Culture Collection (ATCC, Manassas, VA, USA). Human metastatic breast cancer lines MDA-MB-231, MDA-MB-436 and mouse embryonic NIH-3T3 fibroblasts were cultured in DMEM with 10% fetal bovine serum (FBS). Human non-metastatic breast cancer MCF7 cells were cultured in IMEM, 10% FBS and 10 *μ*g/ml insulin. Plasmids were transfected into cells using Amaxa Nucleofector Kits (Lonza Inc., Allendale, NJ, USA), and cells harvested 24 h after transfection were subjected to western blotting analysis and Transwell invasion assays. Transfection efficiency with the control GFP vector system was approximately 70%.

### Plasmid construction and RNAi

Plasmids encoding c-myc-tagged or GFP-Drp1 were gifts from Dr. Quan Chen (Institute of Zoology, Chinese Academy of Sciences, Beijing, China). Drp1 was inserted in pECFP-N1 using *Eco*RI and *Bam*HI. The GBP2 construct cloned from a human fetal liver cDNA library was inserted into the vector pGEX-6P1 (GE Healthcare, Princeton, NJ, USA) to generate pGST-GBP2 using *Bam*HI and *Not*I. This construct was also inserted into the vector pFLAG-CMV-4 (Sigma, St. Louis, MO, USA) to generate pFLAG-GBP2 using *Bgl*II and *Kpn*I and inserted into pEGFP-C2 and pEYFP-C1 (Clontech, Palo Alto, CA, USA) to generate pEGFP-GBP2 or pEYFP-GBP2 using *Kpn*I and *Bam*HI, respectively. The GBP1 construct purchased from GeneCopoeia Company was inserted into the pFLAG-CMV-4 vector (Sigma) to generate pFLAG-GBP1 using *EcoR*I and *Bam*HI. The GBP2 mutant was generated by site-directed mutagenesis using Pfu-ultra poly-merase (Stratagene, La Jolla, CA, USA) followed by DpnI digestion (Fermentas Inc., Glen Burnie, MD, USA) according to the manufacturer’s instructions. Small interfering RNA (siRNA) oligonucleotides were purchased from Dharmacon (Lafayette, CO, USA) with sequences targeting GBP2 (5′-GGAGGUUACCGUCUCUUUA-3′) GBP1 (5′-AGGCAUGUACCAUAAGCUA-3′) or Drp1 (5′-ACUAUUGAAGGAACUGCAAAAUAUA-dAdG-3′). For GBP2, GBP1 or Drp1 shRNA construction, the siRNA was cloned into the pSilencer 2.1-U6 hygro plasmid. The vector expressing GBP2, GBP1, Drp1 shRNA or its scramble were transiently transfected into cells using the Amaxa Nucleofector Kit. Cells were treated with IFN-*γ* (50 ng/ml) and harvested after 48 h for western blotting analysis and Transwell invasion assays. The siRNA-insensitive mutant of GFP-Drp1 was constructed as described previously.^17^

### Antibodies and reagents

Antibodies of Drp1 and Tim23 were purchased from Becton Dickinson (San Jose, CA, USA). Antibodies for Drp1 Ser 637 and Ser 616 were from Cell Signaling Technology Inc. (Beverly, MA, USA). Antibodies for Mfn1, Flag and *β*-actin were from Sigma. The GST antibody was from Novagen (Madison, WI, USA), and the Mfn2 antibody was from Abnova (Taipei, Taiwan). Antibodies for GBP2 and GBP1 were from Sigma. Antibodies were diluted 1 : 100 from the stock concentration for immunostaining and 1 : 1000–1 : 5000 for western blotting. The recombinant human IFN-*γ* was from PeproTech (Rocky Hill, NJ, USA). U0126 was from Cell Signaling Technology. MitoTracker Red and Alexa Fluor 647-, Rhodamine- or fluorescein isothiocyanate (FITC)-conjugated second antibodies were purchased from Invitrogen (Carlsbad, CA, USA).

### GST pull-down assay

GST-tagged GBP2 or GST control was expressed in *Escherichia coli* BL21 (DE3) strain under induction of 1 mM isopropyl-*β*-D-thiogalactopyranoside. The GST-GBP2 and GST protein were purified using GST-bind resin (Novagen) according to manufacturer’s instructions. GST or GST-GBP2 bound to resins was incubated with MDA-MB-231 cell lysate overnight and was then extensively washed with RIPA buffer. Cellular proteins bound to GST or GST-GBP2 were analyzed by SDS-PAGE. Expressed GST protein was used as a control. SDS-PAGE gels were stained using a ProteoSilver Plus Silver Stain Kit (Sigma-Aldrich, St. Louis, MO, USA) according to the manufacturer’s instructions. Bands appearing specifically in the GST-GBP2 precipitate were excised for mass spectrometry.

### Mass spectrometric analysis

All NanoLC-MS/MS analyses were performed on a nanoLC-LTQ-Orbitrap XL mass spectrometer (Thermo, San Jose, CA, USA) at a resolution of 60 000. Solvents used were 0.5% formic acid water solution (buffer A) and 0.5% formic acid acetonitrile solution (buffer B). Trapping was performed at 2 *μ*l/min in buffer A for 15 min, and elution was achieved with a gradient of 0–32% in buffer B for 80 min, 32–50% buffer B for 6 min, 80% buffer B for 6 min at a flow rate of 300 nl/min. Eluted peptide cations were converted to gas-phase ions using Nanospray Flex ion source at 2.0 kV. Raw data were processed using Proteome Discoverer (version 1.4.0.288, Thermo Fischer Scientific, Waltham, MA, USA). MS 2 spectra were queried with the SEQUEST engine against the uniprot human complete proteome database. Database searches were performed with the following parameters: precursor mass tolerance 20 ppm; MS/MS mass tolerance 0.6 Da; two missed cleavage for tryptic peptides; variable modifications oxidation (M), Methylthio (C), and biotin-maleimide (C). Peptide spectral matches were validated by a targeted decoy database search at a 1% false discovery rate. With proteome Discoverer, peptide identifications were grouped into proteins according to the law of parsimony.

### Immunofluorescence and confocal microscopy

Cells on coverslips were fixed with 4% paraformaldehyde in PBS, permeabilized with 0.1% Triton X-100, blocked with 1% BSA and 10% horse serum and incubated with primary antibodies and Rhodamine- or FITC-conjugated secondary antibodies. MitoTracker Red (50 nM; Invitrogen) was used for mitochondrial staining. Images were visualized with an Olympus FV1000 confocal microscope (Olympus, Tokyo, Japan) and processed using the Fluoview software (Olympus). Mitochondrial length and Drp1 localized at mitochondria were measured as described^20^ before using the Image-Pro Plus software (Media Cybernetics, Rockville, MD, USA). The relative abundance of mitochondria in the lamellipodia region was calculated by using the method described before.^17^

### Transwell invasion assays

Matrigel invasion assays were carried out at 37 °C for 16 h using 24-well Transwell inserts (Corning-Costar, Cambridge, MA, USA) coated with 30 *μ*g of Matrigel (BD Biosciences, San Jose, CA, USA). Cells (50 000) suspended in 200 *μ*l of serum-free medium were seeded into the upper chamber and 600 *μ*l of NIH-3T3 CM were placed in the lower chamber. Cells that invaded through the membrane were counted and normalized relative to 10 000 seeded cells. CM from NIH-3T3 cells was collected and used as a chemoattractant as previously reported.^17^

### *In vivo* tumor xenograft experiments

MDA-MB-231 cells were stably transfected with GFP, GFP-GBP2, scramble shRNA or Drp1 shRNA vector, and then transfected cells (1.0 × 10^6^) were injected into the tail vein of 6–8-week-old female athymic nude BALB/c mice. Six weeks later, mice were killed, and the incidence and number of visible lung metastases were recorded. The lungs were dissected, rinsed in PBS and fixed in Bouin’s solution (picric acid: formaldehyde: acetic acid, 15 : 5 : 1), followed by paraffin embedding and sectioning (5-*μ*m thick), stained with hematoxylin and eosin and scanned using a TissueGnostics TissueFAXS Cytometry instrument (TissueGnostics GmbH, Vienna, Austria) to quantify the number of metastatic nodules. All studies involving mice were approved by the Institutional Animal Care and Treatment Committee of Sichuan University.

### FRET assays

All FRET assays were performed using an Olympus FV1000 confocal laser scanning microscope 24 h after cells were transfected. The donor (CFP) was excited at 458 nm, and its fluorescence was detected at 478–498 nm (CFP channel), whereas excitation at 514 nm and emission at 545±15 nm were used for detecting the acceptor (YFP) (YFP channel). FRET was detected at an excitation of 458 nm and emission of 545±15 nm (FRET channel). Fluorescence images of the transfected cells were taken at the CFP, YFP and FRET channels sequentially. Dequenching of the donor fluorescence by photobleaching of the acceptor YFP was performed by illuminating the transfected cells at 514 nm for 250 iterations, and then CFP-Drp1 images were taken in the same focal plane. The FRET efficiency was calculated using the equation *E*=1−(*F*DA/*F*D), where *F*DA and *F*D are the fluorescence intensity of CFP in the cells expressing both donor and acceptor and donor alone (acceptor was quenched), respectively.

### Immunoprecipitation

Cells were collected and washed twice with PBS and re-suspended in lysis buffer (0.75 M aminocaproic acid, 50 mM Bis-Tris, pH 7.0, 1.5% n-dodecyl-b-d-maltopyranoside, 1 mM phenylmethyl sulfonyl fluoride, 1 mg/ml leutpeptin and 1 mg/ml pepstatin) for 30 min on ice. Then samples were centrifuged at 72 000 × *g* for 20 min at 4 °C. The primary monoclonal antibody (2 *μ*g) was added into the supernatant containing 500 *μ*g protein and incubated at 4 °C overnight. After incubation with Protein G-agarose (Millipore, Boston, MA, USA) for 2 h at 4 °C, the beads were washed three times with lysis buffer and boiled with loading buffer for 5 min. Proteins were separated by SDS–polyacrylamide gel electrophoresis, and western blotting was performed with the indicated antibodies.

### Cellular fractionation

Cells were fractionated by differential centrifugation as described previously.^41, 42, 43^ Briefly, cells were harvested and resuspended in three volumes of hypotonic buffer (210 mM sucrose, 70 mM mannitol, 10 mM Hepes, pH 7.4, 1 mM EDTA) containing 1 mM phenylmethylsulfonyl fluoride, 50 mg/ml trypsin inhibitor, 10 mg/ml leupeptin, 5 mg/ml aprotinin and 10 mg/ml pepstatin. After gentle homogenization with a Dounce homogenizer (Kontes, Vineland, NJ, USA), the cell lysates were centrifuged at 1000 × *g* for 5 min to remove unbroken cells and nuclei. The supernatant was collected and centrifuged at 10 000 × *g* to pellet the mitochondria-enriched heavy membrane fraction. The supernatant was further centrifuged at 100 000 × *g* to obtain the cytosolic fraction.

### Assay of cell viability and death

Cell viability was determined by CellTiter-Glo Luminescent Cell Viability Assay from Promega (Madison, WI, USA) as described.^44, 45^ Apoptosis was analyzed using the Annexin V Apoptosis Kit (Calbiochem, Gibbstown, NJ, USA). Briefly, cells were harvested and re-suspended at a concentration of 1.0 × 10^6^ cells per ml and stained with FITC-labeled annexin V and propidium iodide as described.^17, 46^

### Statistical analysis

Results are expressed as the mean±S.E.M. of at least three independent experiments, and statistical comparisons were made using Student’s *t*-test or two-way ANOVA with Bonferroni correction where there were multiple comparisons. *P*<0.05 was considered to be statistically significant.

## Publisher’s Note

Springer Nature remains neutral with regard to jurisdictional claims in published maps and institutional affiliations.
