# Supplementary figures and images for: Guanylate-binding protein 2 regulates Drp1-mediated mitochondrial fission to suppress breast cancer cell invasion
Source: Cell Death Dis. 2017 Oct 26;8(10):e3151–. doi: 10.1038/cddis.2017.559 (PMC5680924; doi:10.1038/cddis.2017.559)

Supplementary fig.-1

**a**

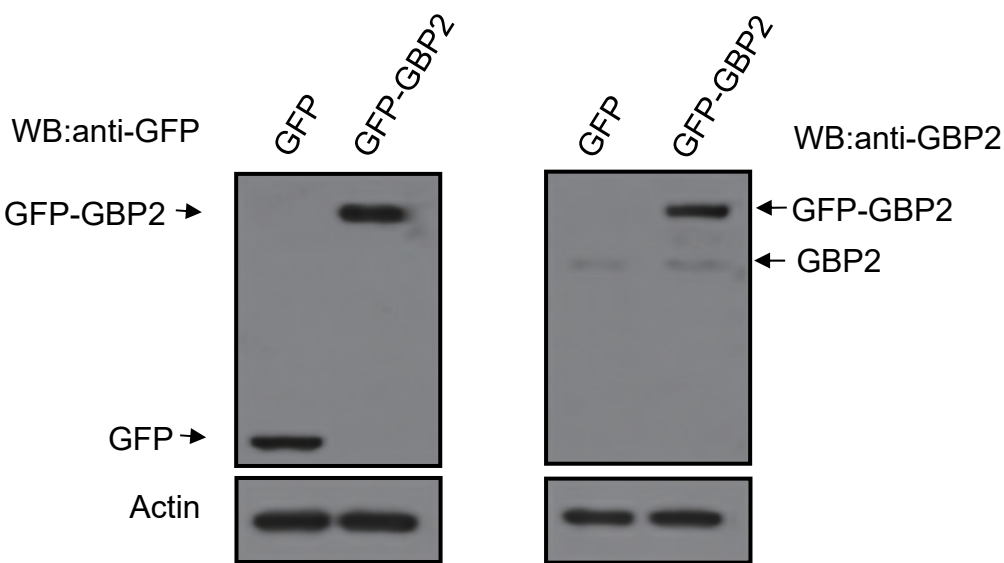

**b**

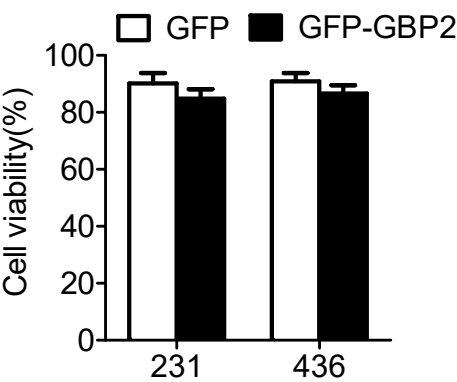

**c**

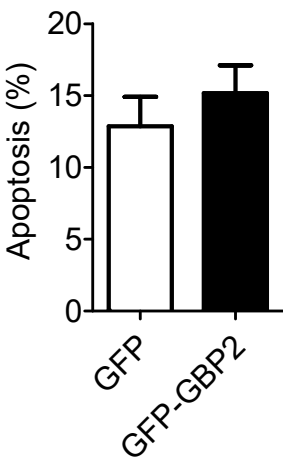

Supplement: Supplementary Figure 1 [file cddis2017559x1.pdf]

Supplementary fig.-2

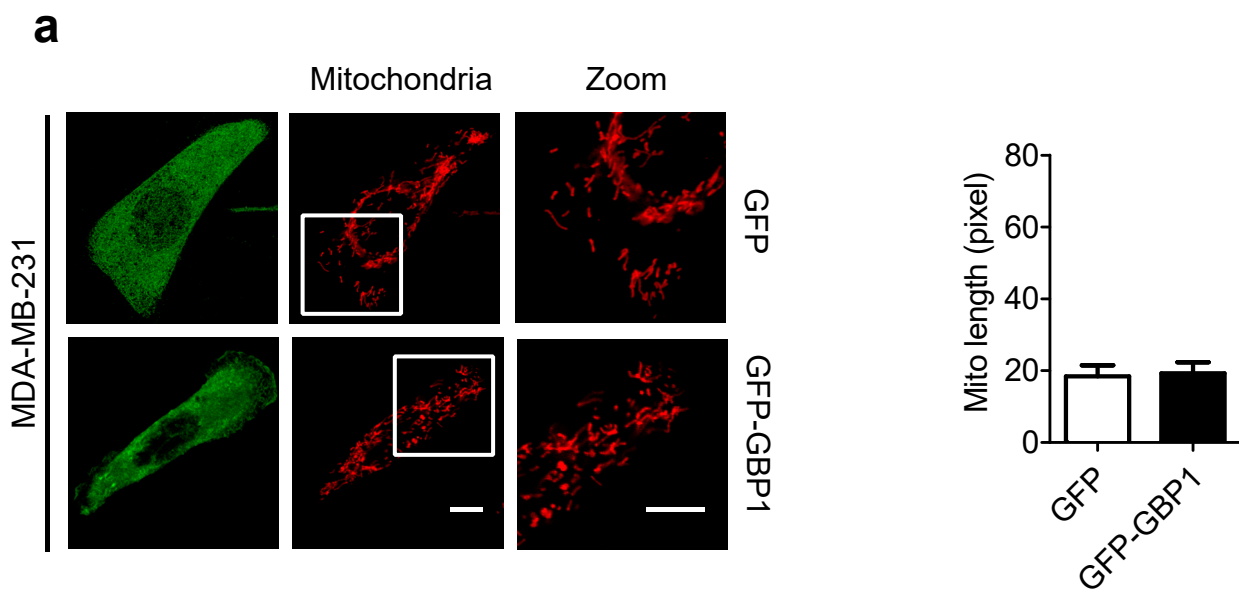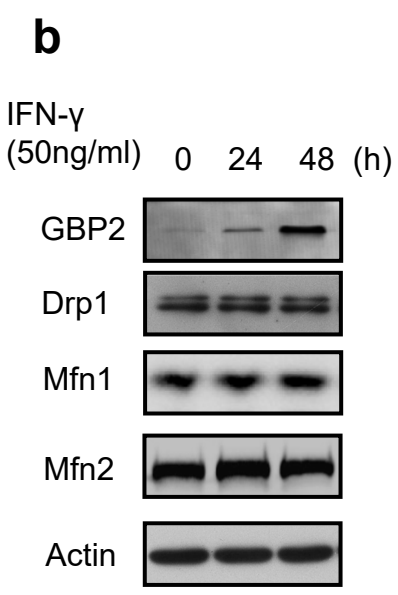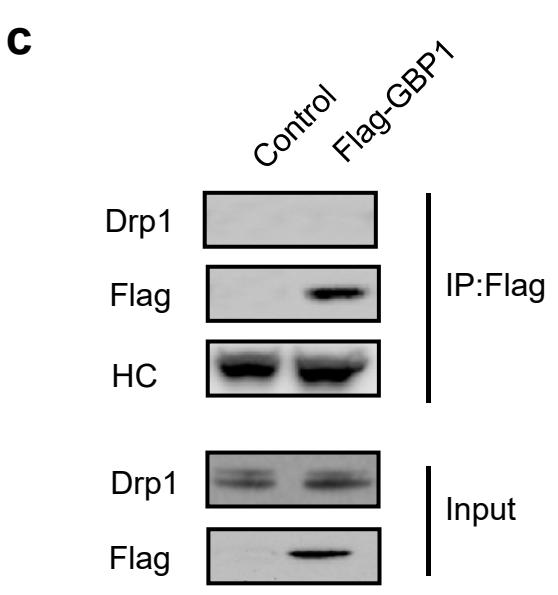

Supplement: Supplementary Figure 2 [file cddis2017559x2.pdf]

## Supplementary fig.-4

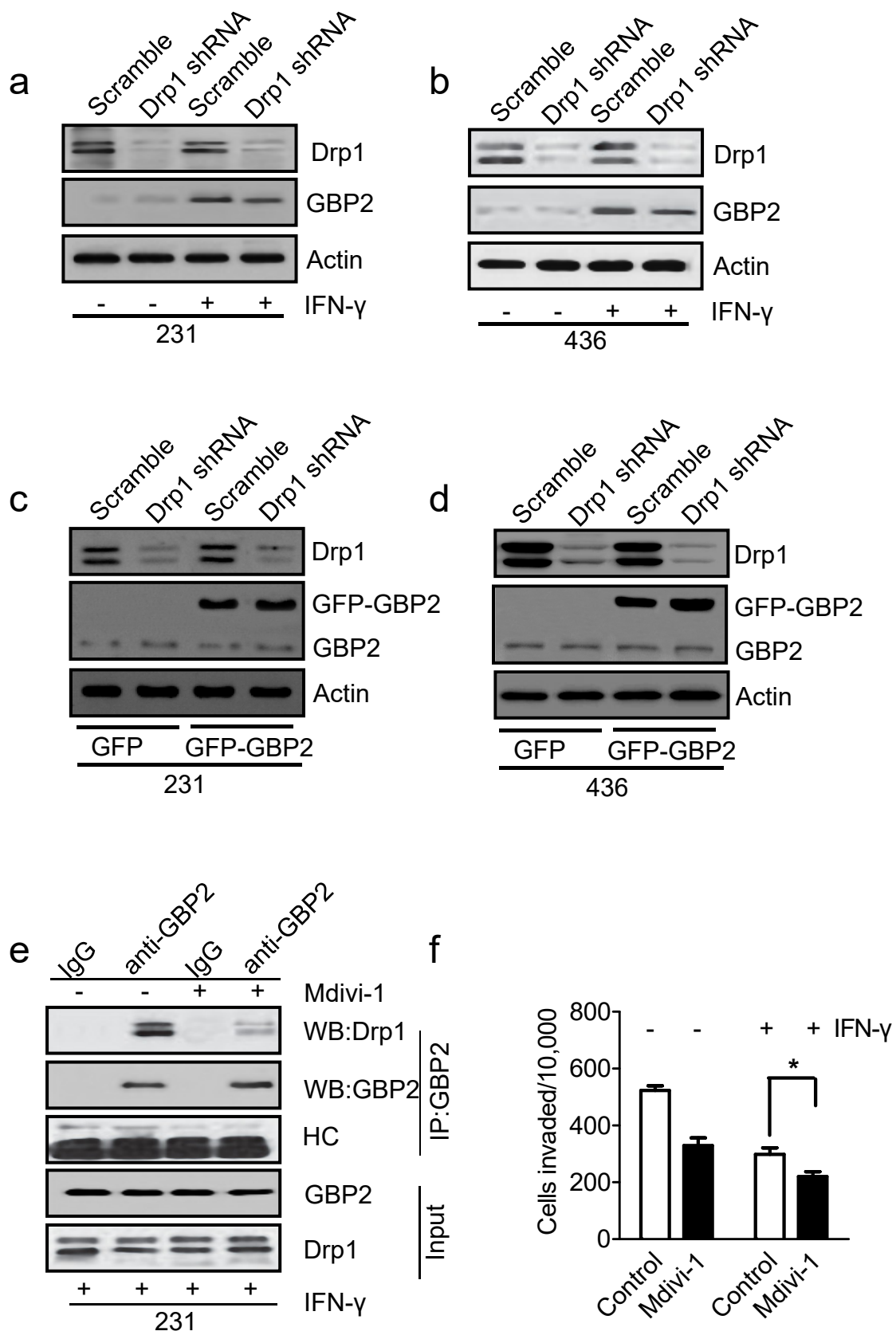

Supplement: Supplementary Figure 4 [file cddis2017559x4.pdf]

Supplementary fig.-5

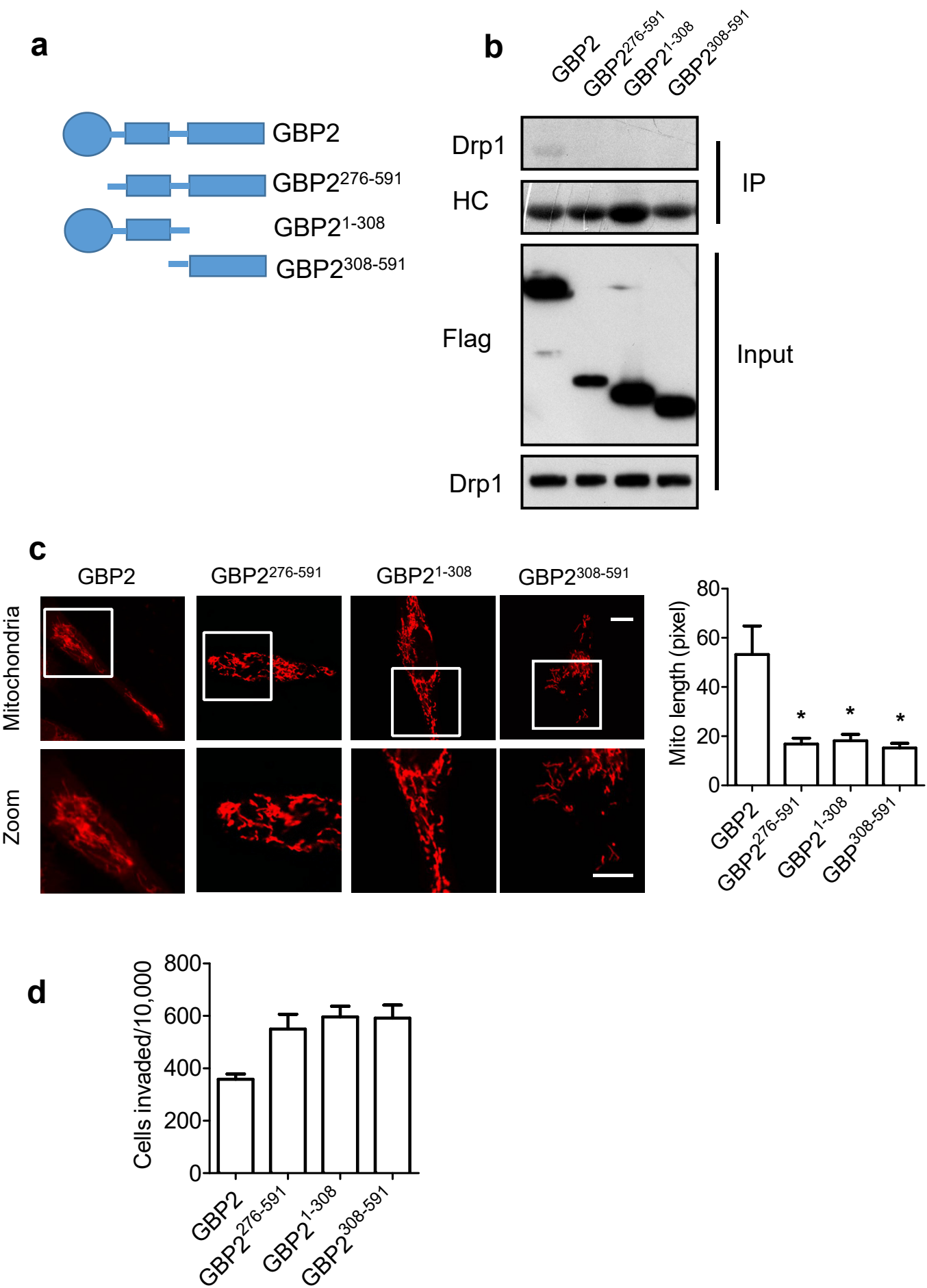

Supplement: Supplementary Figure 5 [file cddis2017559x5.pdf]

Supplementary fig.-6

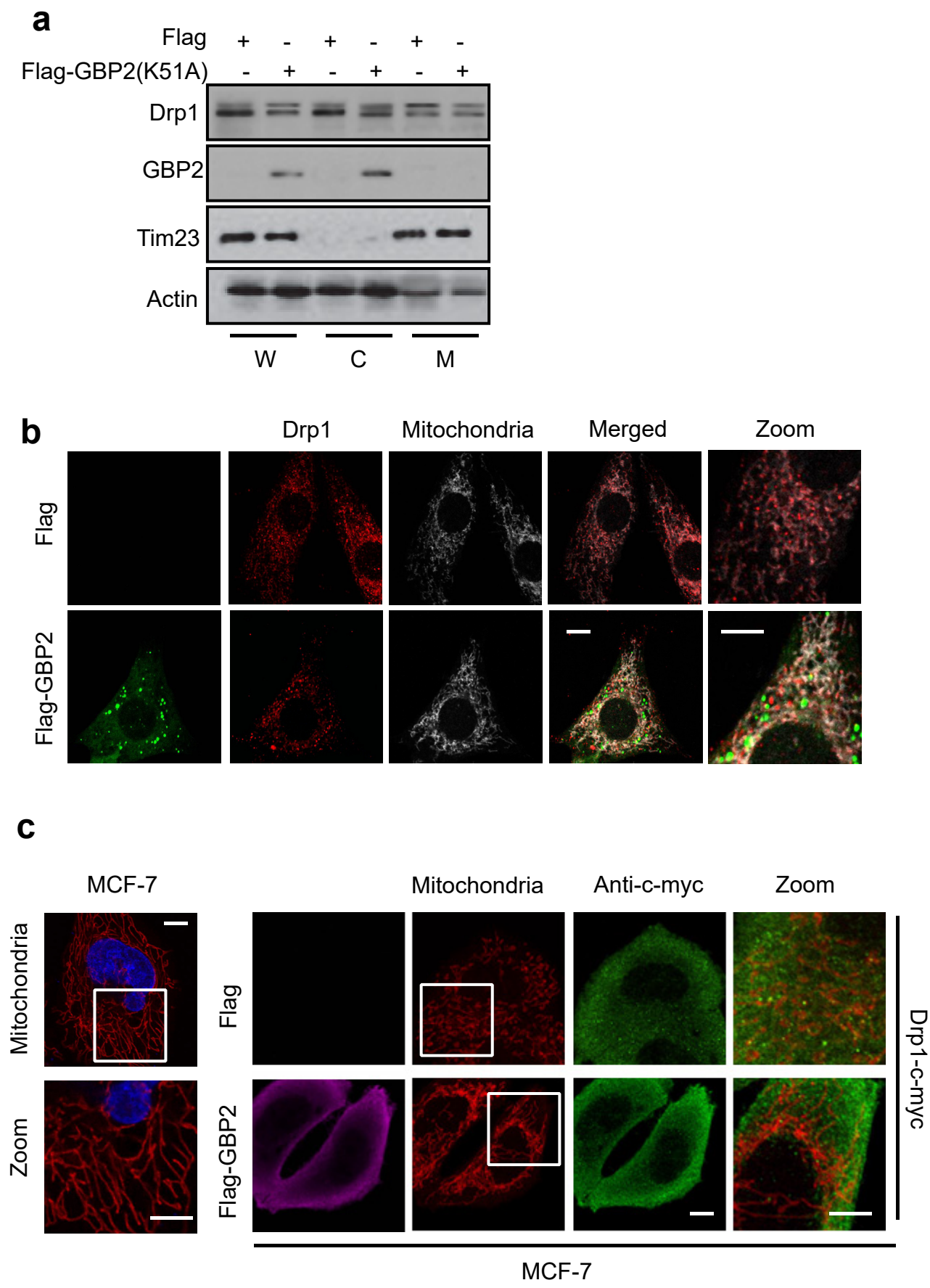

Supplement: Supplementary Figure 6 [file cddis2017559x6.pdf]

Supplementary fig.-7

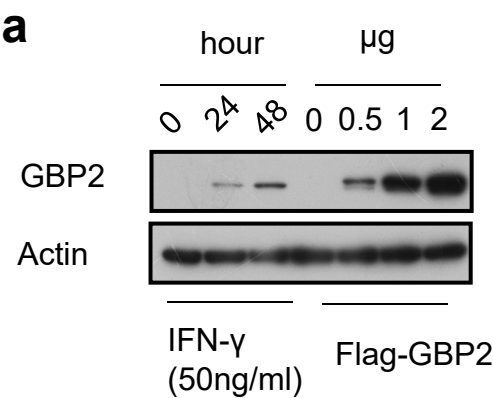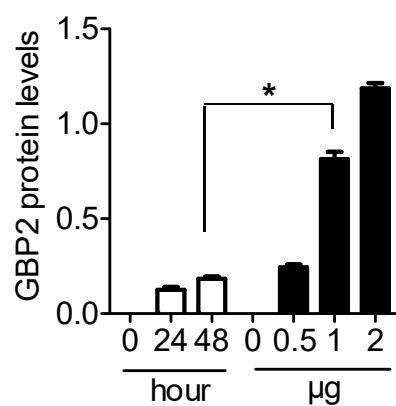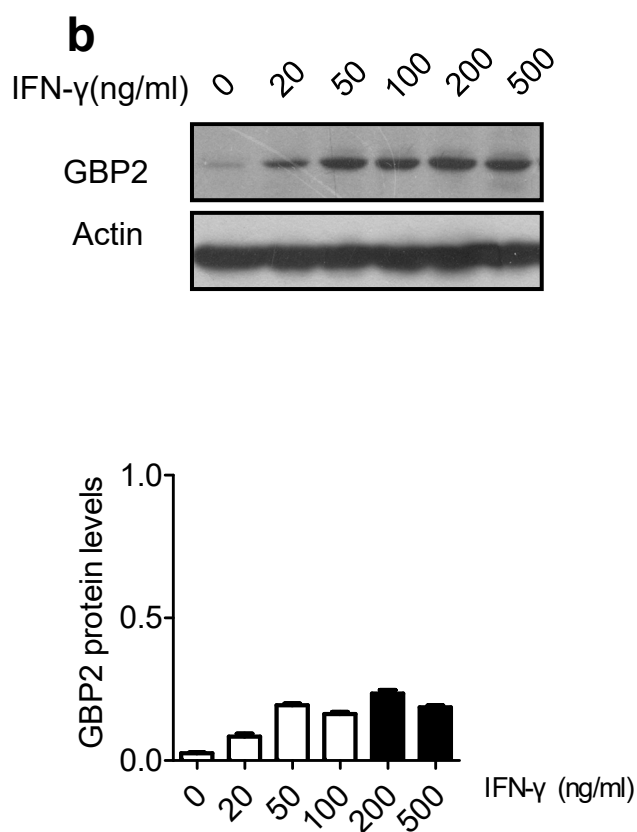

Supplement: Supplementary Figure 7 [file cddis2017559x7.pdf]
