# Supplementary material for: Guanylate-binding protein 2 regulates Drp1-mediated mitochondrial fission to suppress breast cancer cell invasion
Source: Cell Death Dis. 2017 Oct 26;8(10):e3151–. doi: 10.1038/cddis.2017.559 (PMC5680924; doi:10.1038/cddis.2017.559)

Supplementary fig.-3

a

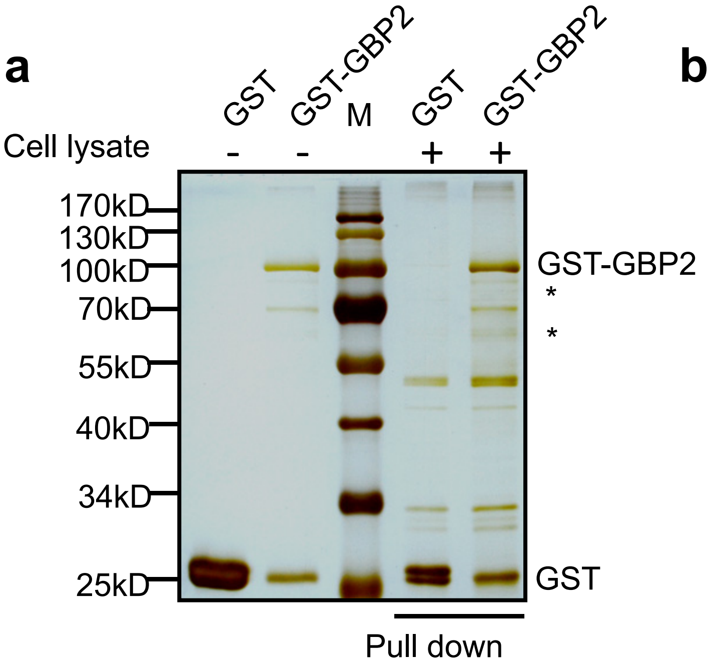

b

Potential binding proteins of GBP-2 analyzed by MALDI-TOF mass spectrometry

**Gene name**	**Protein name**
**CTTN**	Isoform 3 of Src substrate cortactin
**MX2**	Interferon-induced GTP-binding protein Mx2
**DRP1**	Dynamin-related protein 1

c

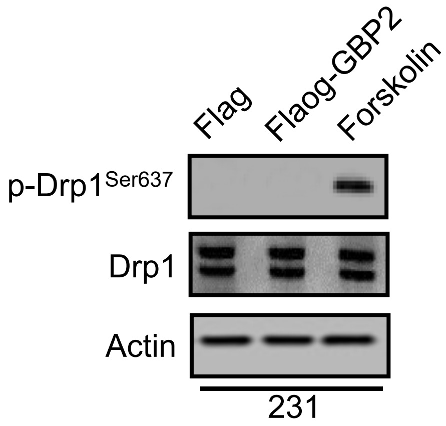

d

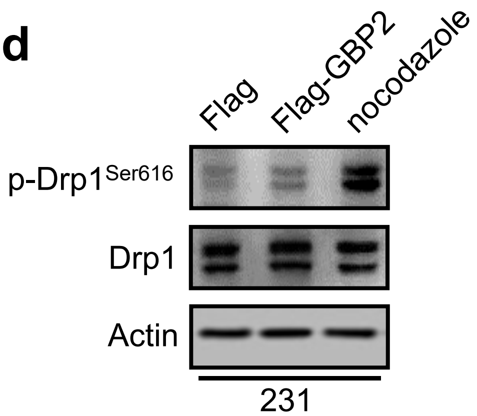

Supplement: Supplementary Figure 3 [file cddis2017559x3.pdf]
