# Supplementary material for: Guanylate-binding protein 2 regulates Drp1-mediated mitochondrial fission to suppress breast cancer cell invasion
Source: Cell Death Dis. 2017 Oct 26;8(10):e3151–. doi: 10.1038/cddis.2017.559 (PMC5680924; doi:10.1038/cddis.2017.559)
Supplement: Supplementary Figure Legends [file cddis2017559x8.docx]

**Supplementary Figure legends**

**Supplementary Figure 1. (a)** GFP or GFP-GBP2 vector were transfected into MDA-MB-231 cells and western blot were analysed by anti-GFP antibody (left panel) or anti-GBP2 antibody (right panel). β-actin was as a loading control. Over-expression of GFP-tagged GBP2 without effects on cell apoptosis in MDA-MB-231 **(b)** or cell viability by CellTiter-Glo Luminescent Cell Viability Assay in MDA-MB-231 and MDA-MB-436 cells **(c)**. Data shown are mean ± s.e.m. (n = 5), *p < 0.05.

**Supplementary Figure 2. (a)** The representative images of MDA-MB-231 cells expressing GFP or GFP-GBP1 (green). Mitochondria were visualized with MitoTracker Red. Right panel is the quantification of mitochondrial lengths in MDA-MB-231. Scale bar, 10 μm. Data shown are mean ± s.e.m. *n* = 22 to 27 randomly selected cells, **p* < 0.05. **(b)** Western blot analysis of expression levels of GBP2, Drp1, Mfn1 and Mfn2 in MDA-MB-231 cells treated with IFN-γ (50 ng/ml) for various time (0-48 h). **(c)** Immunoprecipitation of the extracts from MDA-MB-231 cells expressing Flag or Flag-tagged GBP1 using anti-Flag antibody, followed by western blot analysis with antibodies against the Flag or Drp1. Heavy chain (HC) of anti-Flag antibody was used as loading control.

**Supplementary Figure 3. (a)** Silver staining analysis of GST and GST-GBP2 precipitates of MDA-MB-231 cell lysates. Black asterisks, potential GBP2 interaction proteins; **(b)** Potential GBP2 interaction proteins identified by MALDI-TOF mass spectrometry. Over-expression of GBP2 does little effect on Drp1 phosphorylation. Western blot analysis of GBP2 effect on Ser 637 status **(c)** or Ser 616 status **(d)** of Drp1. 100 ng/ml nocodazole treated cells for 16 hour or 20 uM Forskolin treated MDA-MB-231cells for 1h as positive control, respectively.

**Supplementary Figure 4. (a)** and **(b)** Western blot analysis of Drp1, GBP2 expression in MDA-MB-231cells or MDA-MB-436 cells transfected with scramble or Drp1 shRNA, then treated with IFN-γ. **(c)** and **(d)** Western blot analysis of Drp1, GBP2 expression in the cells described in **(a)** and **(b)**, then co-transfected with GFP-GBP2. **(e)** A selective inhibitor of Drp1, Mdivi-1, inhibits GBP2-Drp1 bindings and migration of breast cancer cells. IFN-γ treated MDA-MB-231 cells for 48 h, and then cells were treated with 25 µM Mdivi-1 (Sigma-Aldrich, St Louis, MO, USA) for 30 min. Treated cells were subjected to subject to immunoprecipitation. **(f)** As described in **(e)**, cells were collected for transwell migration assays in response to NIH-3T3 CM (right panel). *n*=4, mean ± s.e.m. * *p* < 0.05.

**Supplementary Figure 5. (a)** Panel of GBP2 and its mutants. GBP2^276-591^, N-terminal GTPase globular domain deletion mutant; GBP2^1-308^, C-terminal helical domain deletion mutant; GBP2^308-591^, N-terminal GTPase globular and middle domains deletion mutant; **(b)** Cell lysates of MDA-MB-231 cells expressing Flag-GBP2, GBP2^276-591^ ,GBP2^1-308^ or GBP2^308-591^ were subjected to immunoprecipitation with anti-Flag antibody, followed by western blot with Drp1 antibody. The heavy chain of immunoglobulins was used as loading control. **(c)** The confocal images of MDA-MB-231 cells expressing Flag-tagged GBP2, GBP2^276-591^, GBP2^1-308^ or GBP2^308-591^ (left panel). Mitochondria were visualized with Mitotracker red. Scale bar, 10 μm. The length of mitochondria was quantified using Image-Pro Plus software (right panel). Data shown are mean ± s.e.m. of 25-30 randomly selected cells, **p* < 0.05. **(d)** The histogram shows cell invasion of MDA-MB-231 cells expressing Flag-tagged GBP2, GBP2^276-591^ ,GBP2^1-308^ or GBP2^308-591^. Data shown are mean ± s.e.m. (*n*=4), **p* < 0.05.

**Supplementary Figure 6. (a)** Flag or Flag-tagged GBP^K51A^ transfected into cells. Equal amounts of protein of whole cell lysate (W), cytosol (C) and mitochondrial (M) fractions were loaded on SDS-PAGE and analyzed by western blot using anti-Drp1 and GBP2 antibodies. β-Actin was used as a loading control of whole cell lysate and cytosol fraction whereas Tim23 was used as the loading control of mitochondrial fraction. **(b)** The confocal images of MDA-MB-231 with either Flag null vector or Flag-GBP2 (green). Mitochondria were visualized with Mitotracker red, showing the white images. Endogenous Drp1 was stained with red. Scale bar, 10 μm. **(c)** The confocal images of MCF-7 cells expressing Drp1-cmyc (Green) with either Flag null vector or Flag-GBP2 (magenta). Mitochondria were visualized with Mitotracker red. Scale bar, 10 μm. Left panel shows control MCF-7 cells.

**Supplementary Figure 7. (a)** Up panel, MDA-MB-231 cells were treated with IFN-γ (50 ng/ml) for 24 h and 48 h or different concentrations of GBP2 construct for 24 h, and harvested for western blot by anti-GBP2 antibody. β-actin was as a loading control. Down panel, quantification of GBP2 expression. **(b)** MDA-MB-231 cells were treated with IFN-γ of different concentration for 48 h for western blot (up panel) and quantification of GBP2 expression (down panel). Data shown are mean ± s.e.m., **p* < 0.05. *n* = 3.
